# The effect of digital therapeutics intervention on improving hypertension management in adults: a meta-analysis of randomized controlled trial

**DOI:** 10.1038/s41440-024-01892-4

**Published:** 2024-10-23

**Authors:** Lu Liu, Jiayue Guo, Xitong Jiao, Lili You

**Affiliations:** Chinese Academy of Medical Sciences and Peking Union Medical College, School of Health Policy and Management, Beijing, 100005 China

**Keywords:** Hypertension, Digital therapeutics, Blood pressure, Lifestyle, Digital therapy, Health management

## Abstract

Digital therapeutics (DTx) intervention is an emerging therapy for the treatment and long-term management of hypertension. We aim to systematically evaluate the overall effect of DTx intervention on improving hypertension management. The systematic review and meta-analysis of RCTs was conducted and the PubMed, EMBASE, Web of Science, and Cochrane Library were searched to identify eligible RCTs published between Jan 1, 1982 and Sep 10, 2023. Random-effect models were utilized to pool estimates of net changes in systolic blood pressure (BP), diastolic BP, BP control rate, body mass index, weight, waist circumference, and physical activity between the DTx group and control group. 15 RCTs were included with a total of 3789 participants. Compared with the control group, DTx intervention was associated with significant changes in systolic BP, diastolic BP, and BP control rate of –3.75 mmHg(95% CI –5.74 to 1.77), –1.79 mmHg (95% CI –2.81 to –0.77) and 1.47% (95% CI 1.10 to 1.95), respectively. In addition, DTx intervention was statistically significant for improving other risk factors such as lower BMI (−0.5 kg/m2, 95% CI –0.86 to −0.15), increased physical activity (66.73 min/week, 95%CI 49.64 to 83.81), and reduced waist circumference (−2.91 cm, 95% CI −5.15 to −0.66). No difference between groups was demonstrated in weight (*P* = 0.30). Subgroup analyses revealed consistent effects of the change in SBP and DBP across study duration, age, sample size, patient baseline status, and intervention scenario settings(*P* > 0.05). DTx intervention may be useful for lowering BP and long-term management of hypertension. More large-size trials providing evidence on the same product are needed.

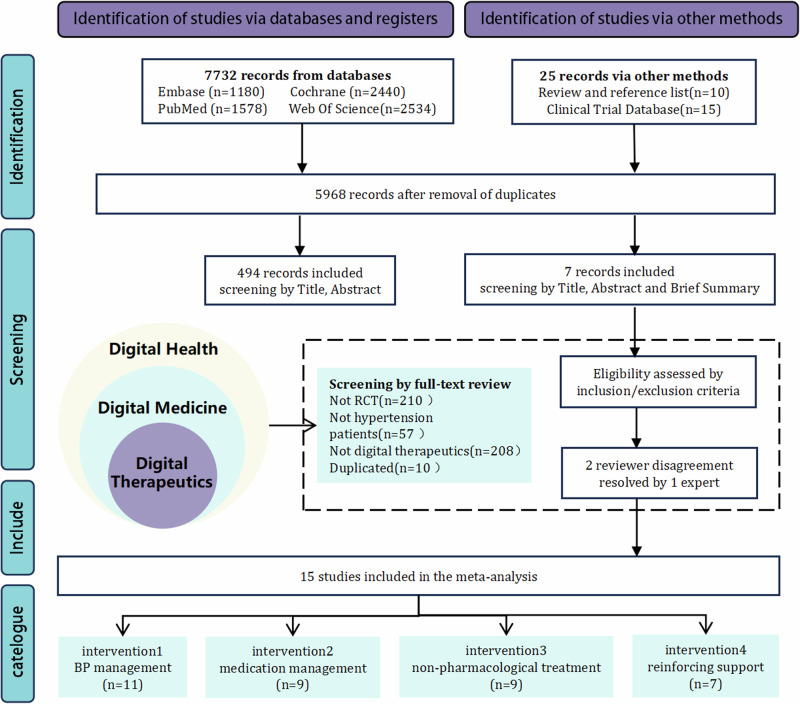

## Introduction

Hypertension is the most common preventable risk factor for cardiovascular disease (including coronary heart disease, heart failure, stroke, myocardial infarction, atrial fibrillation and peripheral artery disease), chronic kidney disease (CKD), and cognitive impairment, and is the leading single contributor to all-cause death and disability worldwide [1, 2]. Hypertension is estimated to affect 33% of adults aged 30–79 worldwide and the number of adults with hypertension doubled from 650 million in 1990 to 1.3 billion in 2019 [3, 4]. Globally, high systolic blood pressure (SBP) leads to 108 million deaths and 235 million disability-adjusted life years [1]. Nevertheless, among adults aged 30–79 years with hypertension, 21% are considered to have their hypertension controlled globally and only 16% in China [5]. Suboptimal adherence, which includes failure to initiate pharmacotherapy, to take medications as often as prescribed, and to persist in therapy long-term, is a well-recognized factor contributing to the poor control of blood pressure in hypertension [6]. In some instances, strategies to promote home blood pressure monitoring(HBPM) and long-term management of hypertension have been shown to reduce blood pressure(BP) and improve hypertension control rates [7, 8], particularly when coupled with interventions that lead to treatment intensification [9].

Mobile health and telemedicine technologies have great potential for development in chronic disease management in recent years [10, 11]. Several review articles evaluated the effectiveness of mHealth or smartphone apps for promoting blood pressure management, medication adherence, and lifestyle improvement [12, 13]. Nevertheless, despite the widespread availability of mobile health interventions and software as a medical device(SaMD) claiming to promote hypertension control or medication adherence, not many products have been developed with the involvement of health professionals, have an independent core program, and have been rigorously validated for BP-lowering efficacy [14, 15]. In 2019, the International Digital Therapeutics Alliance defined digital therapeutics (DTx) as, delivering evidence-based therapeutic interventions that are driven by high-quality software programs to prevent, manage, or treat a medical disorder or disease [16]. Digital therapeutics emphasizes evidence-based interventions and high-quality devices to optimize patient care and health outcomes, further rigorously specifying the techniques and categories of digital interventions used for disease treatment and management. Some randomized controlled trials (RCTs), such as HERB-DH1, demonstrated the efficacy of digital therapeutics systems in reducing BP and improving home self-measurement without medications compared with conventional lifestyle interventions [17, 18]. A 2023 narrative review reported potential BP-lowering mechanisms of DTx in hypertension and the process of treating hypertension with DTx [11]. However, due to the ambiguity of the necessary attributes of DTx and the lack of consistency of diverse interventions of DTx products, the analysis only reported a review of the mhealth app interventions. The evidence on the digital therapeutic approach has not yet been synthesized and appraised and the pooled quantitative effect of DTx in hypertension management remained unclear.

Therefore, the current systematic review and meta-analysis centers on the ability of DTx products to generate and deliver validated and measurable medical effects directly to hypertension patients from RCTs, including lower BP and other relevant characteristics. It also further explores where the effects varied by trial duration, and setting, as well as to identify individuals who might benefit most from digital therapeutics products.

## Methods

This systematic review of RCTs was conducted in accordance with the Cochrane Handbook for Systematic Reviews of Interventions (Version 6.4). The results were reported according to the Preferred Reporting Items for Systematic Reviews and Meta-Analyses Statement (2020). The protocol is registered with PROSPERO (CDR 42024501858).

### Search strategy

The search focused on the identification of studies relating to digital therapeutic for hypertension management conducted worldwide. Medline (via Ovid), Embase, Web of Science, the Cochrane Central Registry of Controlled Trials (CENTRAL), and the Cochrane database of systematic reviews for the period up to September 10, 2023 were searched to identify relevant studies. A combination of search terms was used, including Hypertension; Hypertension or Blood Pressure, High or Blood Pressures, High or High Blood Pressure or High Blood Pressures (for the disease type); Smartphone App, Smartphone Apps, Smartphone application, APP Mobile; Telemedicine, Computer-Assisted Drug therapy, Medical Informatics Application, eHealth, Telecommunication, Mobile health, mhealth, Digital health, Telemonitoring (for digital therapeutic). The full search strategy was included in the Appendix p2. Reference lists from related original articles and reviews were also investigated. No language limitation was applied. To ensure the comprehensiveness of the search, we also scanned clinical research database (https://clinicaltrials.gov/), the product library of the digital therapeutics alliance (https://dtxalliance.org/understanding-dtx/product-library/) and the catalog of cleared and approved medical device information from U.S. Food and Drug Administration (https://www.accessdata.fda.gov/scripts/cdrh/devicesatfda/index.cfm) to obtain current clinical trials and products related to antihypertensive digital therapeutics to look back upon the potentially additional studies.

### Study selection

To determine the studies to be assessed further, two review authors independently scanned the abstract, title, or both sections of every record retrieved. All potentially relevant articles were investigated as full text. Two investigators (JXT and GJY) independently screened all titles and abstracts to determine eligibility for inclusion in the meta-analysis. In cases of discordance of opinion, a third author (LL) was consulted to achieve consensus. We identified studies among adults and only randomized controlled trials by manually reviewing titles, abstracts, and texts from eligible articles.

### Eligibility criteria

Eligible RCTs included the following characteristics:

1. adult patients (≥18 years) with hypertension who were defined: as patients with inadequately controlled blood pressure (BP ≥ 140/90 mmHg or home BP ≥ 135/95 mmHg whether or not they are receiving hypertension medication) and stage 1 hypertension (BP is 130–139 or 80–89 mmHg).
2. software was included if it was used for more than one disease (e.g., diabetes and hypertension) and patients with hypertension or comorbid diseases were included as study participants.
3. the intervention in the experimental group fit into the following intervention criteria for DTx.
4. changes in SBP or diastolic blood pressure (DBP), blood pressure control, and and health related outcomes were reported, excluding articles that reported only outcomes not related to improvements of health management.
5. had a comparison group receiving usual clinical care, health education, advice only, or standard follow-up.

Both peer-reviewed publications and conference abstracts were included, but conference abstracts need to report RCT findings as a complement to the RCT’s research design and rationale articles.

According to the definition and core principles of the DTx Alliance, the criteria for digital therapeutics were explicated in this study as (1) Software driving: the interventions of products need to be computer-based software, platforms, and smartphone applications(apps). Through other channels that are not specifically designed for hypertension management algorithms or programs are not eligible, such as telephone, WeChat, SMS, and e-mail, etc (2) Algorithm and model supporting: independent algorithms or models for digital therapeutic products need to be designed for or developed with the collaboration of medical professionals (3) Intervention: software or apps that respond to user input and aim to generate tailored content to blood pressure control or other multiple hypertension care behaviors improvement domains through feedback, customized antihypertensive plans (including dynamically adjusted medication, lifestyle, and disease care prescriptions and advice), reinforcement and rewards, risk scoring and alerts, patient decision support, goal setting, or reminders. Any program, website, or app that is used only for hypertension monitoring or self-reporting, without a feedback interactive system, only for communication or general health education between patients and professionals, and only targeted exclusively at health professionals is not eligible.

### Data extraction

For studies that fulfilled inclusion criteria, three review authors (LL, GJY, JXT) independently extracted relevant population and intervention characteristics using standard data extraction templates and completed an intervention description and information extraction form. This included (1) Author(year); country; (2) Sample size; (3) Age (year); (4) Setting; (5) Study population; (6) BP Change as Primary Outcome; (7) SBP/DBP at Baseline; (8) Information related to primary and secondary outcomes; (9) Intervention duration(month), and (10) Adherence and participation measures. Any disagreements were resolved through discussion. Post-intervention blood pressure change means and standard deviations were recorded whenever possible.

### Outcomes and quality assessment of included studies

The revised Cochrane risk-of-bias version 2 tool was used to assess the quality of the studies on aspects of selection (random-sequence generation and allocation concealment); performance and detection (masking of participants, personnel, and assessors; deviations from intended interventions; missing outcome data; and measurement of the outcome); appropriateness of analysis (selection of the reported outcome); and bias arising from period and carryover effects (for crossover studies). For quality assessment, 2 coders independently assessed the quality of included studies (LL and GJY).

Integrating all the factors contributing to hypertension management into a unified model to describe how they might affect outcomes is challenging. As many of the health outcomes take many years to develop, it is not practical to use them as primary outcome measures for this review as followed-up in the studies would not be long enough to demonstrate differences in these. However, more proximal variables such as changes in SBP and DBP, and blood pressure control rate, may change over suitable scales. The prespecified primary outcomes of interest were average changes in SBP, DBP, and BP control rate, and secondary outcomes included BMI, weight, waist circumference, and physical activity.

### Statistical analysis

The Synthesis Manager used a random-effects model and computed mean difference (MD) to generate pooled estimates of outcomes between the intervention group and the control group. Heterogeneity was assessed by the Q and I^2^ statistics. If data were available from two or more studies, the outcomes were included in the meta-analysis.

For each research included, the net effect size was defined as the difference in BP change between the intervention and control groups, and was calculated by subtracting the baseline (a) to follow-up (b) change in the control group from the corresponding change in the intervention arm: (I_a_−I_b_) − (C_a_−C_b_). If SE or 95% CI were reported instead of SD or SE, then these were calculated as described in Chapter 7.7.3.2 of Cochrane’s handbook [19]. If none of SD, SE, or 95% CI could be obtained from published data or following communication with the authors, then SDs were imputed according to the recommendations in Chapter 16.1.3.1 of Cochrane’s handbook [19].

Priori-defined subgroup analyses were performed to further evaluate the effect of DTx on BP control according to hypertensive status at baseline (whether inclusion criteria included inadequate BP control), antihypertensive use, intervention duration (<6 months or 6 ~ 12 months or ≥12 months), age (<60 years or ≥ 60 years) and intervention setting (hospitals or primary care clinics), sample size (<200 or ≥200 participants). Inadequate BP control was defined as an in-office BP ≥140/90 mmHg or a HBPM ≥135/85 mmHg, per the JNC-8 guideline (Paul A. James et al.) [20] and 2017 ACC/AHA BP Guideline (Whelton et al.) [21]. The associated *p*-value of <0.1 suggests that heterogeneity is unlikely to be caused by chance alone.

The robustness of summary effect size was assessed via sensitivity analyses that included sequentially removing each study and reanalyzing the remaining datasets to identify if a single study was responsible for the direction of associations. Potential publication bias between studies was assessed by visually inspecting a funnel plot of the mean change in SBP and DBP plotted against their corresponding SE. The Egger test was used to quantify any asymmetry among the funnel plots, with *p* < 0.05 indicating potential bias and *p* > 0.05 indicating no significant publication bias. Statistical analyses were performed using Review Manager 5.4 (The Cochrane Collaboration, 2020, Copenhagen, Denmark) and Stata 17.

## Results

### Study selection and characteristics

**Fig. 1:**
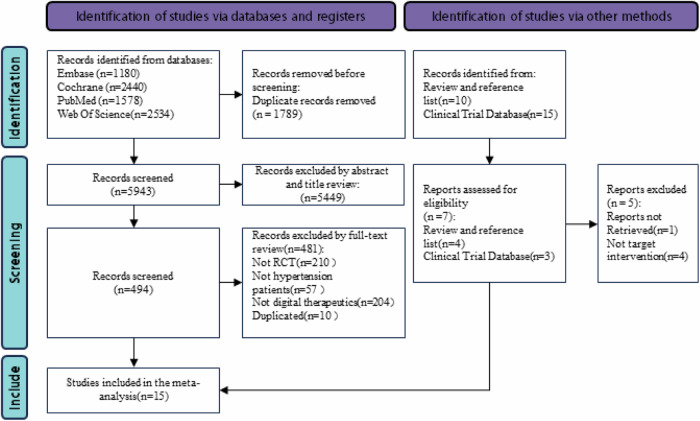
Preferred reporting items for systematic reviews and meta-analyses (PRISMA) flowchart of study selection

A total of 7732 records were imported into Endnote and 1789 duplicates were removed. After title and abstract screening, of these 5943 articles, 5449 were removed because they did not meet the inclusion criteria. 494 were used for full-text screening. A total of three authors first worked independently in the screening and selection process and then compared their results. Disagreements were resolved through a round of discussion.15 studies with a total of 3789 participants fulfilled the inclusion criteria and were included in the current meta-analysis. (Fig. 1)

Characteristics of the included studies are shown in Table 1. 12 studies were conducted in the past 5 years (2019 and after) and 3 between 2011 and 2018. 7 studies were undertaken in the United States [12, 22–27], 2 in China [28, 29], 1 in Japan [17], 1 in Spain [30], 1 in England [18], 1 in German [31], 1 in Iran [32], 1 in Korea [33]. 13 RCTs employed a parallel group design and 2 a cluster design [23, 31]. 13 studies used smartphone APP as the primary mode of delivery of the digital health intervention, 1 used website [26], and 1 used software accessed via wireless tablet computers [27]. All studies had a mean of 253 participants and 66.7% retention over a mean duration of 6 months. At baseline, participants had a mean age of 56.4 years, a mean SBP of 146.2 mmHg, and a mean DBP of 88.4 mmHg.

**Table 1:** Study design and baseline characteristics of randomized controlled trials of digital therapeutic intervention for hypertension

Author (year); country	Sample size, n	Age (year)	Setting	Study population	Other diseases	BP Change as Primary Outcome	SBP/DBP at Baseline		Outcomes		Duration	Intervention			
							SBP	DBP	Primary	Secondary		1	2	3	4
Stephen D Persell (2020); USA [24]	333	58.9 (12.8)	primary care clinics	Uncontrolled HT	Asthma or COPD, diabetes, coronary heart disease, stroke, heart failure	Yes	140.6 ± 12.2 mmHg (intervention) 141.8 ± 13.4 mmHg (control)	89.4 ± 8.7 mmHg (intervention) 89.2 ± 9.2 mmHg (control)	▲SBP −8.3 ± 13.8 mmHg (intervention), −6.8 ± 13.7 mmHg (control), intervention vs. control, *p* = 0.16; ▲DBP-4.3 ± 84 mmHg (intervention), −3.6 ± 9.5 mmHg (control), intervention vs. control *p* = 0.61；▲Rates of controlled BP^a^ 72 (50.0%, intervention), 78 (51.3%, control)	▲Self-reported physical activity (at least moderate exercise) 26.7 min/week (−5.4, 58.8), *p* = 0.10; ▲Mean self-confidence in controlling BP score (5-point scale) 0.36 (0.18, 0.54), *p* < 0.001	6mo	✓	✓	✓	
Caroline Nespolo David (2023); USA [12, 40]	**231**	55.4 (9.5)	hospital	Uncontrolled HT + AM	Diabetes	No	143.7 ± 11.4 mmHg (all) 143.5 ± 11.4 mmHg (intervention) 144.5 ± 11.3 mmHg (control)	89.6 ± 8.1 mmHg (all) 89.2 ± 8.0 mmHg (intervention) 91.3 ± 8.1 mmHg (control)	▲Weight(kg) RR = −0.39 (−1.49, 0.70); ▲No smoking RR = 0.99 (0.92,1.07); ▲Physical activity ≥ 150 min/week RR = 1.21 (1.03, 1.42); ▲Moderate or no alcohol intake ≤100 g (women)/≤200 g(men)/week RR = 1.12 (1.00, 1.25); ▲ Following ≥ two of six dietary recommendations RR = 1.22 (1.04, 1.42)	▲Body fat mass between-groups difference −4.05 kg (−8.14, −0.03), intervention vs. control *p* = 0.052; ▲BMI between-groups difference −1.56 kg/m^2^ (−3.49, −0.37), intervention vs. control *p* = 0.113; ▲Waist circumference between-groups difference −4.36 cm (−8.81, −0.082), intervention vs. control *p* = 0.054	6mo	✓		✓	
Yue Ma (2022); China [28]	**210**	60.6 (10)	community health service centers	HT + AM	Heart disease, diabetes, arthritis, cervical spondylosis, stroke, herniated lumbar discs	Yes	149.7 ± 12.36 mmHg (intervention) 150.59 ± 11.09 mmHg (control)	93.77 ± 7.4 mmHg (intervention) 93.25 ± 7.11 mmHg (control)	▲SBP-11.74 ± 14.34 mmHg (intervention), −1.01 ± 11 mmHg (control), intervention vs. control *p* = 0.005; ▲DBP −5.53 ± 4.95 mmHg (intervention), 1.69 ± 5.14 mmHg (control), intervention vs. control *p* = 0.005; ▲Proportion of participants with controlled BP^a^−17.14% (intervention), −1.15% (control), p = 0.145	▲Body weight group*time interaction effect between groups −1.71 kg (−2.23, −1.18), *p* = 0.0005; ▲BMI group*time interaction effect between groups −0.59 kg/m2 (−0.77, −0.41), *p* = 0.0005; ▲Waist circumference group*time interaction effect between groups −3.19 cm (−3.79, −2.58), *p* = 0.0005; ▲The self-care behavior, motivation and self-efficacy (possible range 0–80) (behavior: MD = 6.38, *p* < 0.001; motivation: MD = 5.85, *p* < 0.001; self-efficacy: MD = 7.13, *p* < 0.001)	3mo	✓		✓	✓
Kazuomi Kario (2021); Japan [17, 41]	**390**	52.4 (8.1) 52.0 (7.6)	hospital	HT + non- AM^b^	Dyslipidemia, diabetes mellitus, proteinuria, nonvalvular atrial fibrillation	YES	144.9 ± 10.4 mmHg (intervention) 144.3 ± 10.4 mmHg (control)	95.0 ± 8.2 mmHg (intervention) 94.3 ± 7.2 mmHg (control)	▲24 h ambulatory SBP −4.9 ± 16.34 mmHg (intervention), −2.5 ± 20.61 mmHg (control), intervention vs. control, *p* = 0.024	▲ Morning home SBP −4.3 mmHg (−6.7, −1.9), intervention vs. control *p* < 0.001; ▲Evening home SBP −3.3 mmHg (−5.8, −0.7), intervention vs. control *p* = 0.013; ▲ Office SBP −3.6 mmHg (−6.2, −1.0), intervention vs. control *p* = 0.006; ▲ Reductions from baseline in ambulatory, home and office DBP and heart rate were also significantly greater in the DTx and control group; ▲Proportion of morning home BP < 135/85 mmHg 22.2% (intervention), 10.4% (control)	3mo	✓		✓	✓
Jessica Chandler (2019); Spain [30, 42]	**56**	46.5 (9.9)	hospital	Uncontrolled HT + AM	Diabetes	Yes	152.3 mmHg (intervention) 150.7 mmHg (control)	86.8 mmHg (intervention) 84.6 mmHg (control)	▲SBP-31.1 mmHg (intervention), −11.8 mmHg (control), intervention vs. control, *p* < 0.01; ▲DBP −12.6 mmHg (intervention),−5.2 mmHg (control), intervention vs. control *p* < 0.01; ▲Percentage of SBP < 140 mmHg −14.4% (intervention), −15.6% (control), *p* = 0.009; ▲Percentage of DBP < 90 mmHg −38.5% (intervention), −30.5% (control), *p* = 0.145	▲Medication adherence^c^ 0.49 (SMASH), 3.39 (control), SMASH vs. control p < 0.001	6mo		✓		
McManus RJ (2020); UK [18]	**553**	66 (10.2)	clinics	Uncontrolled HT + AM	NO	Yes	151.7 ± 11.8 mmHg (intervention) 151.6 ± 11.1 mmHg (control)	86.4 ± 9.6 mmHg (intervention) 85.3 ± 9.9 mmHg (control)	▲SBP −13.3 ± 9.65 mmHg (intervention), −9.79 ± 10.34 mmHg (control), intervention vs. control *p* < 0.05；▲DBP −6.2 ± 6.24 mmHg (intervention), −5.5 ± 6.32 mmHg (control), intervention vs. control *p* < 0.05	▲Weight loss 29/243 (11.9%, intervention), 57/251 (22.7%, control), *p* = 0.002; ▲Dose changes in AM RR = 2.0 (1.5, 2.7); ▲Type changes in AM RR = 1.5 (1.1,1.9); ▲MA *p* = 0.97; ▲Patient enablement difference in PEI^d^ −0.4 (−0.5, −0.2)	12mo	✓	✓	✓	
Frauke Leupold (2023); German [31]	**525**	59.4 (9.7)	clinics	Uncontrolled HT + AM	NO	Yes	156.9 ± 14.8 mmHg (all) 157.8 ± 16.2 mmHg (intervention) 155.9 ± 13.1 mmHg (control)	93.7 ± 9.6 mmHg (all) 94.8 ± 9.8 mmHg (intervention) 92.5 ± 9.3 mmHg (control)	▲SBP −22.5 ± 10.37 mmHg (intervention), −13.9 ± 10.87 mmHg (control), intervention vs. control *p* < 0.05; ▲DBP −10.5 ± 6.13 mmHg (intervention), −9.2 ± 6.41 mmHg (control), intervention vs. control *p* < 0.05；▲ Proportion of BP target range^a^ 62.6% (intervention), 44.6%(control), *p* < 0.001	▲ Number of inpatient treatments 23 (8.7%, intervention), 35 (13.5%, control), *p* = 0.1; ▲Number of serious cardiovascular events (hospitalizations)^e^ 12(4.5%, intervention), 9 (3.5%, control), *p* = 0.14; ▲Patients’ satisfaction with BP treatment 89.4% (intervention), 79.5% (control), *p* < 0.001; ▲Number of antihypertensives 0.33 (intervention), 0.15 (control), *p* = 0.37 at baseline, *p* = 0.001 at follow up; ▲Utilization of PIA-ICT^f^ 10.59 ± 11.25 (min-max 0–48) medication plans were transferred to patients; 249.79 ± 228.90 (min-max 0–1138) blood pressure readings were transmitted from patients to practices; 3.71 ± 7.95 (min-max 0–91) chats were sent from patients to practices; practices sent 6.93 ± 8.87 (min-max 0–49) messages; ▲Satisfaction with the PIA-Intervention (five-point scale: 1=very good to 5=poor) 1.76 ± 2.00 (patients), 1.8 ± 0.50 (general practices)	12mo	✓	✓		✓
LaPrincess C (2022); USA [23, 43]	**85**	54.2 (14.3)	community	HT	Cardiovascular diseases: coronary heart disease, stroke, heart failure	NO	NR	NR	▲Change in mean LS7 score 1.9 ± 1.9 (intervention), 0.7 ± 1.9 (control), RR = 1.13 (0.62, 1.65), intervention vs. control *p* < 0.0001; ▲Feasibility of intervention (Health-ITUES score) 4.2 ± 0.7 points (0.7)	▲Healthy diet score (range 0–5) 0.79 ± 0.86 points (intervention), −0.08 ± 1.19 points (control), RR = 0.87 (0.54, 1.21), *p* < 0.0001; ▲ Physical activity 219.70 ± 396.73 min/week (intervention), 76.09 ± 286.67 (control), RR = 143.61 (7.67, 279.56), *p* = 0.04; ▲BMI 0.16 ± 1.30 kg/m^2^ (intervention), 0.14 ± 2.24 kg/m^2^ (control), RR = −0.30 (−1.23,0.62), *p* = 0.52; ▲SBP −3.09 ± 11.61 mmHg (intervention), −7.25 ± 13.14 mmHg (control), RR = 4.16 (−1.38, 9.70), p < 0.05; ▲DBP −0.53 ± 8.27 mmHg (intervention), −2.5 ± 11.85 mmHg (control), RR = 1.97 (−2.38, 6.31), *p* < 0.05; ▲Total cholesterol −10.96 ± 25.8 mg/dL(intervention), −17.74 ± 25.93 mg/dL(control), RR = 6.77 (−6.81, 20.36), *p* = 0.33; ▲Fasting glucose 2.89 ± 18.44 mg/dL (intervention), 6.73 ± 10.64 mg/dL (control), RR = −3.84 (−11.73, 4.05), *p* = 0.34	6mo			✓	✓
Kyle Morawski (2018); USA [22]	**411**	52.0 (-)	primary care /community	Uncontrolled HT + AM	No	Yes	151.4 ± 9.0 mmHg (intervention) 151.3 ± 9.4 mmHg (control)	NR	▲SBP − 10.6 ± 16.0 mmHg (intervention), −10.1 ± 15.4 mmHg (control), *p* = 0.97;▲MA (MMAS-8^g^) 0.4 ± 1.5 points (intervention), −0.01 ± 1.5 points (control), *p* < 0.01	▲Proportion of controlled BP^a^ 67 (35.8%, intervention), 69 (37.9%, control), *p* = 0.34	3mo	✓	✓		
Ali Bozorgi (2021); Iran [32]	**120**	52.0 51.6	hospital	HT	No	No	108.1 ± 13.5 mmHg (intervention) 114.9 ± 14.3 mmHg (control)	NR	▲MA (Hill-Bone Checklist) 65.1 (65.04–65.23, intervention), 59.7 (59.60–20.36, control) at 24th week, mean change in intervention group 5.9 points (5.03, 6.69);	▲Healthy diet adherence to low-fat diet (range 1–20) 1.7 (1.30–2.10) points, adherence to low-salt diet(range 1–20) 1.5 (1.16–1.90) points; ▲ BMI mean change 1.2 (0.77, 3.2) kg/m^2^; ▲MAP^h^ mean change 3.4(1.6, 5.2) mmHg;▲Moderate physical activity (0–300 min/week) mean change 100.0 (61.7, 138.3) min/week; ▲The predisposing factors of adherence to treatment (knowledge, attitude, and self-efficacy) 2.9 points (1.6–4.2) in knowledge, 2.3 points (1.2–3.4) in attitude, 1.7 points (1.3–2.2) in self-efficacy;▲Satisfaction and usability of the app 18.41 (min 16, max 20)	2mo	✓	✓	✓	✓
Ke Gong (2020); China [29]	**443**	59.27 (7.4) 58.20 (7.5)	hospital	HT	No	Yes	141.19 ± 10.12 mmHg (intervention) 140.51 ± 10.44 mmHg (control)	82.59 ± 9.621 mmHg (intervention) 83.89 ± 8.618 mmHg (control)	▲SBP −8.99 ± 6.415 mmHg (intervention), −8.99 ± 6.415 mmHg (control), intervention vs. control *p* < 0.05; ▲DBP −7.04 ± 6.135 mmHg (intervention), −4.14 ± 8.213 mmHg (control), intervention vs. control *p* < 0.05; ▲Percentage of controlled BP^i^ −38% (intervention), −28% (control), *p* = 0.00	▲Low medication adherence improvement§ 48 (21%, intervention), 18 (13%, control), p = 0.004	6mo	✓	✓		✓
Patricia J. Neafsey (2011); USA [27]	**160**	68.6 (8.7)	primary care	HT + AM	Chronic Disease	No	128.3 ± 14.6 mmHg (all) 129.1 ± 15.5 mmHg (intervention) 127.4 ± 13.4 mmHg (control)	74.5 ± 9.5 mmHg (all) 74.27 ± 8.88 mmHg (control) 74.7 ± 10.1 mmHg (intervention)	▲Patient Adverse Self-Medication Behavior Risk Score (Anderson and Spencer 2002) −4.5 (intervention), −2.4 (control), intervention vs. control *p* = 0.033	▲SBP −2.6 mmHg (intervention), 1.1 mmHg (control), change between groups was non-significant;▲DBP 2.0 mmHg (intervention), 0 mmHg (control), change between groups was non-significant; ▲OTC-Rx Knowledge Score −0.5(control), 4.2 (intervention), *p* < 0.05; ▲OTC-Rx self-efficacy 0.2 (control), 0.8 (intervention), *p* < 0.05; ▲Satisfaction with the PEP-NG between groups was 0.2, *p* = 0.0431	3mo		✓		
Dong-Ju Choi (2022); South Korea [33, 34]	**173**	59.8	tertiary hospitals	Uncontrolled HT + AM	NO	Yes	148.9 ± 8.4 mmHg (intervention) 150.0 ± 8.6 mmHg (control)	NR	▲Home SBP −20.0 ± 13.5 mmHg (SMBP-App) vs. −14.9 ± 12.9 mmHg (only SMBP), SMBP-App vs. only SMBP *p* = 0.012	▲MA95.2% (intervention),90.4%(control), *p* = 0.004; ▲Proportion of adherence over 95% 72/88 (intervention), 46/85 (control), *p* = 0.01	6mo	✓	✓		
Lesli E. Skolarus (2018); USA [26]	**73**	58 (9.8)	community (churches)	Uncontrolled HT	NR	No	160.7 ± 23.6 mmHg (intervention) 162.2 ± 20.5 mmHg (control)	99.0 ± 11.8 mmHg (intervention) 99.2 ± 17.8 mmHg (control)	▲Feasibility of the Reach Out processes 47% of participants texted back BP, 26% responded with their BP every week, 100% reported satisfaction with the intervention	▲SBP −11.3 ± 22.9 mmHg (intervention), −14.4 ± 26.4 mmHg (control), *p* = 0.6; ▲DBP −8.6 ± 15.9 mm Hg (intervention), −9.5 ± 12.9 mmHg (control), *p* = 0.79	6mo	✓		✓	✓
Jessica Chandler (2020); USA [25]	**26**	46.5 (13.0) 43.4 (14.2)	community and clinics	Stage 1 HT^j^	No	Yes	133.2 mmHg (intervention) 132.0 mmHg (control)	75.1 mmHg (intervention) 77.4 mmHg (control)	ΔSBP −11.6 mmHg (intervention), −0.2 mmHg (control), *p* < 0.04	▲DBP −6.4 mmHg (intervention), 2.4 mmHg (control), *p* < 0.04; ▲Percentage of participants with SBP < 130 mmHg 60.3% (Tension Tamer), 35.8% (control), *p* = 0.003; ▲ Proportion of participants meeting 75% adherence benchmark 38.5% (Tension Tamer), 27.3% (control), *p* = 0.582	12mo			✓	

*HT* hypertension, *AM* antihypertensive medications, *NR* not reported, *RR* relative risk, *MD* mean difference, *MA* medication adherence, *ICT* information and communication technology, *PEP-NG* “next generation” of the Personal Education Program, *SMASH* Smartphone Med Adherence Stops Hypertension, *LS7* Life’s Simple 7 score (range 0–14), *Health-ITUES* Health Information Technology Usability Evaluation Scale (range 1–5)

Intervention1: blood pressure management (blood pressure self-monitoring, recording, goal setting, reminders, and abnormal blood pressure alerts)

Intervention2: medication management (medication reminder; medication adherence measurement; medication change reminder; medication tailored strategies)

Intervention3: non-pharmacological treatment (lifestyle changes: healthy diet, reducing salt intake, reducing alcohol and cigarette consumption, weight control, physical activity, improving sleep conditions, coping with stress)

Intervention4: reinforcing support (providing incentives to help maintain self-care behaviors; community discussions on self-efficacy, self-regulation, social support, and barriers/facilitators to healthy lifestyles)

^a^Defined as BP < 140/90 mmHg

^b^No use of antihypertensive medication for ≥3 months prior to enrollment

^c^Measured using medication tray timestamped data, 1 point is awarded for a 3-h window of the predesignated time, 0.5 points within a 3–6 h window, and 0 points for more than 6 h.

^d^Modified patient enablement instrument (PEI) is scored from 1 to 7, with lower scores implying higher enablement

^e^Serious cardiovascular events included stroke, myocardial infarction, blood pressure derailing, heart failure, renal failure, and death

^f^Personal computer-supported case management of hypertensive patients to implement guideline-based hypertension therapy using a physician-defined and -supervised, patient-specific therapeutic algorithm

^g^Morisky Medication Adherence (MMAS) Scale score < 6

^h^(Mean arterial pressure) ${{{\rm{MAP}}}}=\frac{{{{\rm{SBP}}}} \,+ \, 2({{{\rm{DBP}}}})}{3}$ mmHg

^i^Hypertensive patients with chronic kidney disease or diabetes should be under 130/80 mmHg, hypertensive patients who are aged 65 years or older should be less than 150/90 mmHg, and other patients should be less than 140/90 mmHg (The 2010 Guidelines for Hypertension Prevention and Treatment in China)

^j^Stage 1 HT is 130–139 mmHg or 80–89 mmHg

We included studies of people with hypertension, but the condition of the participants varied from study to study. For instance, five studies involved patients with hypertension who also had diabetes mellitus [12, 17, 24, 28, 30]. Seven studies included patients with various other diseases, such as coronary heart disease, stroke, heart failure, asthma, etc. One study did not report on any other conditions in the participants [26], and seven studies focused exclusively on patients with primary hypertension who did not have any other diseases [18, 22, 25, 29, 31, 32, 34]. The participants were already taking antihypertensive medications at baseline in nine studies. Only one study included non-medicated participants [17], and in 6 studies most participants were not receiving antihypertensives. 8 RCTs only included participants with uncontrolled hypertension, whereas 7 RCTs also included participants with controlled hypertension, with 1 RCT included participants with Stage 1 hypertension [32]. 9 studies were conducted with patients recruited from primary care clinics or the community, and 6 were conducted in hospitals. The follow-up period ranged from 8 weeks to 12 months.

Intervention types varied considerably. The intervention categories in the eleven studies involved hypertension monitoring, recording, reminders, and abnormal blood pressure alerts [12, 17, 18, 22, 24, 26, 29–33]. The carriers of 24 h ambulatory BP monitoring (ABPM) are HPCP APP [24], HERB APP [17], PIA APP [31], Medisafe APP [22] automatic oscilloscope equipment [12], Bluetooth monitor [30, 33]. Patients can also manually record their BP [12, 23, 29], and the APP can give feedback on blood pressure level on the graphic icon [32], and display weekly or monthly blood pressure change charts [30]. Some interventions offered telemonitoring via online data recording forms and had the ability to notify general practitioners about atypical readings. Automatic prescription generation without the need to see a general practitioner was also used. The app alerts users if there is a problem with their blood pressure, users can check information about high blood pressure through the app [29, 34].

Four studies included medication reminder [22, 24, 29, 32], providing alerts to remind patients when they need to take their medications and generate medication adherence reports based on BP or a list of medications and their preferred dosing times from patients. One included medication adherence measurement [30], automatically selecting from the information base to tailor the feedback to the user based on the medication adherence (MA) grading. One included medication change reminder [18], prescribers were asked via email to implement pre-planned medication changes when the average home BP was above target. Three included tailored medication strategies, and embedded algorithms that analyze the input medication data and generate recommendations for the user [27, 31, 33].

Apps were also used for self-care like generating self-care plans, and health reports to help hypertensive patients improve and maintain self-care behaviors [17, 28]. Interventionists managed weekly shared board posts to solicit discussions on self-efficacy, self-regulation, social support, and barriers/facilitators to healthy lifestyles [23]. Due to the smartphones’ capabilities, apps included features reinforcing support, providing advice by “virtual nurses” based on biological, psychological, social data [17], and saving user-recorded information in a web portal for physicians and researchers [32]. Additional functionality included emergency care: Users can call the emergency number with one click to get timely medical treatment [29].

Nine studies assessed the effect of the intervention on promoting a healthy diet and reducing salt intake [17, 26, 32], reducing alcohol and cigarette consumption [17], weight control [12, 17, 24, 26, 28, 32], physical activities [26], improving sleep conditions [17, 24], coping with stress [17, 25], and providing cessation incentives [32]. DTx delivered evidence-based health information to patients about hypertension management or online education sessions, ranging in frequency from once a day to once a week. Some interventions allowed participants to interact with health professionals via face-to-face communication. Two studies assessed the feasibility of apps [23, 26].

Most studies were designed to control groups with usual care or enhanced usual care, (eg, laboratory tests and ancillary clinical services tailored to the condition). In most studies, the control group received education only at baseline; however, some studies provided ongoing health education [18, 27]. One study allowed control participants to delay access to the DTx intervention [23]. Three studies required patients to conduct self-monitoring of BP (SMBP) [29, 34], and one provided a BP tracking app with home monitoring [24].

### Relative effects of the intervention

**Fig. 2:**
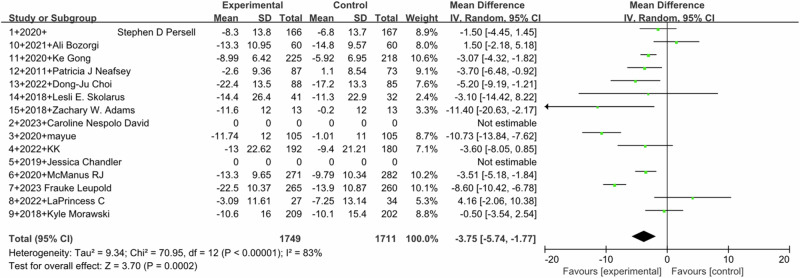
Forest plot for the effect of DTx intervention on improvement in SBP

**Fig. 3:**
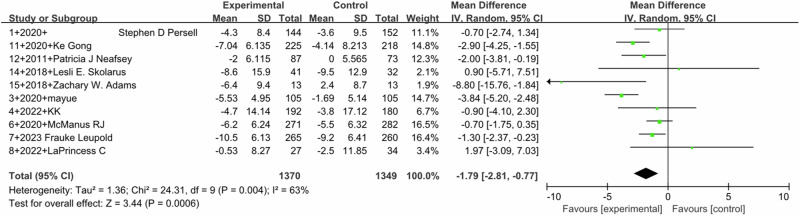
Forest plot for the effect of DTx intervention on improvement in

**Fig. 4:**
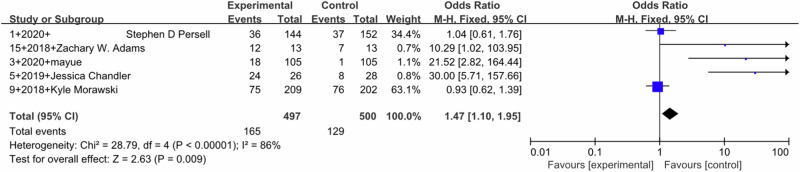
Forest plot for the effect of DTx intervention on improvement in BP control rate

Pooled outcomes of SBP (Fig. 2) and DBP (Fig. 3) were similar. SBP (MD –3.75, 95% CI –5.74 to −1.77; 13 studies) with high heterogeneity (I^2^ = 83%) and DBP (MD –1.79, 95%CI –2.81 to –0.77; 10 studies) with moderate heterogeneity (I^2^ = 63%) both showed a significant effect in favor of the intervention (*P* < 0.0001). Five studies reported the BP control rates, and pooled analysis showed a statistically significant effect of the intervention (MD 1.47, 95% CI 1.10–1.95, I^2^ = 86%). (Fig. 4)

Five studies reported BMI and pooled analysis showed a statistically significant effect of the intervention (MD −0.50 kg/m^2^, 95% CI −0.86 to −0.15, *P* = 0.005) (Fig. S1). Two studies reported weight and pooled analysis showed no effect of the intervention (MD −0.90 kg, 95%CI −2.62 to −0.81, *P* = 0.30) (Fig. S2). Two studies reported waist circumference and pooled analysis showed a moderate effect of the intervention (MD −2.91 cm, 95% CI −5.15 to −0.66, *P* = 0.01) (Fig. S3). Physical activity is measured in minutes of physical activity per week. Three studies reported physical activity and pooled analysis showed moderate effect of the intervention (MD 66.73 min/week, 95% CI 49.64 to 83.81, *P* < 0.001) (Fig. S4). Details were available in Appendix p3.

### Subgroup analysis

Subgroup analysis showed that whether the blood pressure of the included population was under control or not, the length of the intervention, the number of follow-ups, the place of the intervention, the age, and whether the test population was taking antihypertensive medication were analyzed as subgroup factors, and none of the differences between subgroups were statistically significant. According to our main findings, the effects of digital therapy on SBP and DBP were all significant (*P* < 0.05), however, after subgrouping, we found that some subgroups did not have satisfactory antihypertensive effects. Also, we hypothesized factors that had an impact on heterogeneity from the subgroup analysis. As shown in the table, when DBP was used as the outcome, after grouping the trial population by whether their blood pressure was controlled or not, the within-group heterogeneity of both subgroups was not significant (*P* > 0.05, I^2^ < 50%), which proved that whether the blood pressure of the trial population was controlled or not was one of the factors influencing the heterogeneity. (Table 2)

**Table 2:** Results from subgroup analyses of the net change in SBP and DBP

Subgroup Analysis	SBP						DBP					
	No. of Studies	Net Change, mmHg	95% Cl	heterogeneity		*P*	No. of Studies	Net Change, mmHg	95% Cl	heterogeneity		*P*
				*I*^*2*^, %	*P* Value					*I*^*2*^, %	*P* Value	
Duration of intervention												
≥6 months	8	−3.97	−6.38 to −1.57	82	<0.00001	0.81	7	−1.39	−2.53 to −0.26	55	0.04	0.23
<6 months	5	−3.4	−7.41 to 0.60	86	<0.00001		3	−2.61	−4.28 to −0.95	53	0.12	
Intervention scenario setting												
hospital	4	−2.59	−4.97 to −0.21	57	0.07	0.37	8	−1.66	−2.88 to −0.43	67	0.004	0.45
primary care clinics	9	−4.28	−7.06 to −1.50	85	<0.00001		2	−2.45	−4.09 to −0.81	21	0.26	
Average age												
≥60 years	4	−5.59	−8.66 to −2.52	79	0.003	0.17	3	−2.15	−4.17 to −0.14	84	0.002	0.62
<60years	9	−2.67	−5.41 to 0.07	85	<0.00001		7	−1.55	−2.83 to −0.26	47	0.08	
Total follow-up												
≥200	7	−4.49	−6.93 to −2.06	88	<0.00001	0.34	5	−1.38	−2.26 to −0.51	43	0.14	0.40
<200	6	−2.38	−5.95 to 1.19	67	0.01		5	−2.43	−4.70 to −0.16	61	0.04	
Take antihypertensive drugs at baseline												
Yes	6	−4.56	−7.95 to −1.18	90	<0.00001	0.35	3	−1.9	−3.62 to −0.17	85	0.001	0.88
No	7	−2.75	−4.48 to −1.02	37	0.14		7	−1.72	−3.11 to −0.33	43	0.11	
Uncontrolled blood pressure at baseline												
Yes	7	−4.4	−7.26 to −1.53	82	<0.00001	0.51	5	−1.06	−2.06 to −0.06	31	0.21	0.09
No	6	−2.98	−6.09 to 0.13	83	<0.00001		5	−2.47	−3.73 to −1.21	49	0.1	

### Sensitivity analysis and bias results

The funnel plot of BP change estimates was roughly symmetrical (Figs. S5 and S6), indicating no significant publication bias (the Egger test: *p* = *0.599* for SBP and *p* = *0.737* for DBP). Six and three terms exceeded the confidence interval respectively, suggesting heterogeneity between studies. Due to the considerable heterogeneity of included studies on the effects of digital therapeutic interventions on SBP and DBP, sensitivity analyses were needed to assess the stability and confidence of the results. The results showed that excluding either study, the combined point estimates of the MDs of the remaining studies were within the 95% confidence interval of the effect size, and the results were not highly variable, with good robustness. (Figs. S7 and S8)

Details of the risk of bias in the included studies have been summarized in Figs. S9 and S10. All of the included studies were randomized controlled trials but none were blinded. Three studies did not implement blinding in outcome assessment [26, 27, 32], and three studies did not implement allocation concealment [25, 26, 30]. Studies mostly generated a low risk of bias, though, in a small number of studies, a comprehensive bias assessment revealed several unclear risk components.

## Discussion

### Principal results

To our knowledge, this study presents the first synthesis of the pooled effectiveness of different DTx interventions in reducing BP and improving other risk factors. Strengths of our study include the use of rigorous standard methodology as documented in the PRISMA and Cochrane guidelines; the comprehensive series of sensitivity analyses performed to ensure the robustness of the calculated summary effect size; and the inclusion of implementation metrics, by limiting inclusion to only randomized controlled trials, thereby restricting potential confounders.

Of the 5943 records identified, 15 RCTs (*n* = 3789 participants) were included in the meta-analysis, that digital therapeutic interventions were more effective in reducing BP (both SBP and DBP) and improving BP control than usual care. These effects were generally consistent across the different trial settings, sample sizes, intervention durations, and among various population subgroups, which suggested a wide target of hypertensive patients of DTx intervention. In addition, DTx was clinically and statistically significant for improving lifestyle-related metrics such as lower BMI, weight loss, increased physical activity, and reduced waist circumference, compared to traditional interventions. Smartphone APPs are the primary mode of delivering digital health therapy interventions (13 studies, 87%), and therefore smartphone-based DTx interventions are becoming increasingly common ways to support medication adherence and management of hypertension [35]. Our findings provided evidence that DTx intervention could be an important strategy for promoting BP control and improving hypertension health management. From the standpoint of the control group, the mode was not only the usual care and clinical treatment, but also included other modes such as self-monitoring of blood pressure (SMBP) [24, 29, 33], health coaching [18, 27], and so on. Thus, there provided even stronger evidence of the superiority of the digital therapeutic intervention versus the conventional intervention in terms of lowering BP and improvement in the effects of risk factors.

The reductions in BP and improving hypertension-related risk factors were clinically important. Considered overall, digital therapeutic intervention had an incremental BP-lowering effect of −3.75 mm Hg (95% CI −5.74 to −1.77) in SBP, and −1.79 mm Hg (95% CI −2.81 to −0.77) in DBP, and 1.47% in BP control rate (95% CI 1.10–1.95%) compared with control groups. Although the overall net effect was modest from an individual perspective, it is important to note that even relatively small reductions in BP can dramatically reduce the incidence of cardiovascular disease and mortality [36]. There were also statistically significant reductions for BMI (−0.50, 95% CI −0.86 to-0.15), weight (−0.90, 95%CI −2.62 to −0.81), and waist circumference (−2.91, 95% CI −5.15 to −0.66) and increasing for minutes of physical activity per week (66.73, 95% CI 49.64 to 83.81). Many meta-analysis found that weight loss and exercise could benefit blood pressure [37, 38], and therefore DTx may be a potentially effective tool to improve outcomes by these intermediary factors among hypertensive patients broadly.

Combining the results of subgroup analyses, it is indicated that DTx interventions were associated with statistically significant BP reductions, regardless of the mode of delivery of the intervention and patient characteristics at baseline. However, this conclusion is tempered by the considerable heterogeneity of included studies and the high risk of bias in most. For age, most study populations (14 studies) had a mean age of 50 years or above, and only one study had a mean age of 46.5 years [25]. The BP-lowering effect caused by DTx may not be meaningful in more segmented age layers for older adults. It may not be meaningful to explore the antihypertensive effects caused by DTx in more segmented age layers for older adults. For the trial sizes, there was no significant difference in BP change between the follow-up population of ≥200 and the population of <200. Possibly, the study with the largest number of hypertensive patients included 553, which is not large enough compared to other hypertensive RCT trials, so the difference is not revealed.

Notably, this study only explored the effect of DTx interventions on hypertension management and did not refine the effect on patients with hypertension only and also with other diseases. The baseline status of the populations included in the study was not quite the same, though studies were all conducted among the general hypertensive population. Most of studies excluded people with grade III hypertension and above or those with other serious conditions, so DTx interventions may be more appropriate for the general uncontrolled hypertension patients. At the same time, only the HERB trial, excluded the intervention of taking medication, and the other studies included populations that were required to take at least one antihypertensive medication or did not include taking medication in the inclusion criteria, so DTx interventions may be used in the future more as an adjunctive therapy in conjunction with pharmacologic interventions in the treatment and management of hypertension.

Evidence from a large individual trial that meets the optimal sample size would be superior to the results from a systematic review of a similar total sample size to detect the effects of treatment [39]. More large-size trials are needed to validate the role of DTx as a comprehensive intervention strategy in the management of hypertension. Our study found that intervention modes varied widely across DTx products, and it is difficult to carry out combined analyses of different delivery modes. Additional RCT studies providing evidence on the same product are needed, only one effectiveness evidence for one DTx product is much inadequate. In the meanwhile, there was a high heterogeneity of these studies. We ran a series of heterogeneity analyses to explore potential sources, such as subgrouping the studies, but could not fully elucidate the reasons. An inherent source of heterogeneity in our study was the inclusion of DTx interventions because these interventions themselves are heterogeneous. Therefore, additional studies need to describe the mediators and moderators of the effectiveness and implementation of these DTx interventions in detail, and focus on the development of universally applicable and consistent strategies, to both further improve their effectiveness as well as increase their availability and feasibility.

### Limitations

This review has some limitations in the evidence. First, all of the included studies were randomized controlled trials but none were blinded, the level of evidence was of moderate quality only. Second, we identified studies from high-income and upper-middle-income countries, primarily, meaning that the findings cannot be generalized. Although lower-middle-income and low-income-countries had higher incidences and debt of hypertension, it is important for future research to collect evidence in these settings too due to the known healthcare equity disparities. Furthermore, considering the availability of mobile phones and the Internet, it is particularly important for patients in rural and remote areas. The effectiveness of these interventions in additional lower-income countries as well as in remote regions warrants examination. Third, 15 RCTs analyzed include those with varying types of blood pressure measurements as primary endpoints, HBPM was the main measurement in the most studies. We used a pooled estimate of the difference in blood pressure before and after the DTx intervention to decrease bias, and tested through sensitivity analysis that removing 24-h ambulatory BP or in-office BP from a single study did not significantly affect the results of the study.

## Conclusions

DTx intervention may be useful for lowering BP and long-term management of general hypertensive adults. More large-size trials and studies providing evidence on the same product are needed to validate the role of DTx as a comprehensive intervention strategy in the management of hypertension.

## Supplementary information

Multimedia Appendix
